# Model development of dose and volume predictors for esophagitis induced during chemoradiotherapy for lung cancer as a step towards radiobiological treatment planning

**DOI:** 10.1186/s12890-023-02667-2

**Published:** 2023-10-09

**Authors:** Rui He, William N. Duggar, Claus Chunli Yang, Srinivasan Vijayakumar

**Affiliations:** Department of Radiation Oncology, University of Mississippi Medical Center, 350 West Woodrow Wilson Ave. Suite 1600, Jackson, MS 39216 USA

**Keywords:** Lung cancer, Esophagitis, Dosimetric parameters, Chemo-radiotherapy

## Abstract

**Background:**

Currently, radiation therapy treatment planning system intends biological optimization that relies heavily upon plan metrics from tumor control probability (TCP) and normal tissue complication probability (NTCP) modeling. Implementation and expansion of TCP and NTCP models with alternative data is an important step towards reliable radiobiological treatment planning. In this retrospective single institution study, the treatment charts of 139 lung cancer patients treated with chemo-radiotherapy were reviewed and correlated dosimetric predictors with the incidence of esophagitis and established NTCP model of esophagitis grade 1 and 2 for lung cancer patients.

**Methods:**

Esophagus is an organ at risk (OAR) in lung cancer radiotherapy (RT). Esophagitis is a common toxicity induced by RT. In this study, dose volume parameters V_x_ (V_x_: percentage esophageal volume receiving ≥ x Gy) and mean esophagus dose (MED) as quantitative dose-volume metrics, the esophagitis grade 1 and 2 as endpoints, were reviewed and derived from the treatment planning system and the electronic medical record system. Statistical analysis of binary logistic regression and probit were performed to have correlated the probability of grade 1 and 2 esophagitis to MED and V_x_. IBM SPSS software version 24 at 5% significant level (α = 0.05) was used in the statistical analysis.

**Results:**

The probabilities of incidence of grade 1 and 2 esophagitis proportionally increased with increasing the values of V_x_ and MED. V_20_, V_30_, V_40_, V_50_ and MED are statistically significant good dosimetric predictors of esophagitis grade 1. 50% incidence probability (TD_50_) of MED for grade 1 and 2 esophagitis were determined. Lyman Kutcher Burman model parameters, such as, n, m and TD_50_, were fitted and compared with other published findings. Furthermore, the sigmoid shaped dose responding curve between probability of esophagitis grade 1 and MED were generated respecting to races, gender, age and smoking status.

**Conclusions:**

V_20_, V_30_, V_40_ and V_50_ were added onto Quantitative Analysis of Normal Tissue Effects in the clinic, or QUANTEC group’s dose constrains of V_35_, V_50_, V_70_ and MED. Our findings may be useful as both validation of 3-Dimensional planning era models and also additional clinical guidelines in treatment planning and plan evaluation using radiobiology optimization.

## Background

Since the late 1980s, radiation therapy (RT) has been considered a standard component of care for patients with lung cancer [1]. The aim of radiation therapy is to achieve an uncomplicated loco-regional control of cancer [2]. The esophagus is one of the critical organs at risk (OAR) when lung cancer is treated by radiation or chemo-radiation therapy. Acute radiation-induced esophageal toxicity is a significant complication during lung cancer treatment [3]. The severe esophagitis can cause patient hospitalization, and breaks in treatment, which will reduce the tumor control probabilities. The late esophageal complication may induce stricture formation that is an abnormal narrowing of a bodily passage, and ulceration, etc. It is therefore prudent to understand and model the dosimetric parameters of the esophagus to esophagitis in order to prevent or reduce the incidence of esophagitis and improve the quality of life of the patients.

Dosimetric goals based on the Dose Volume Histogram (DVH), such as those presented by QUANTEC [4, 5], gained popularity relatively, however, their correlation to actual clinical outcomes is often ambiguous in daily clinical practice [6]. Normal tissue complication probability (NTCP) modeling is one way to optimize a radiation treatment plan and predict the risk of radiation injury to normal tissue. In fact, incorporation of tumor control probability (TCP) and NTCP modeling into radiation therapy treatment planning is highlighted by AAPM Task group 166 [6, 7] as a huge opportunity on the frontier of clinical radiotherapy. Recently RaySearch has implemented various published NTCP models into their treatment planning system (TPS)-RayStation and RayBiology for biology optimization purpose, but the practical use of this optimization methodology is not well understood nor seen as reliable at this time [8]. The purpose of this retrospective study is to correlate the dosimetric dose-volume parameters of esophagus with incidence of esophagitis, and to establish a dose predictive model to predict the incidence of esophagitis for lung cancer chemoradiotherapy (chemo-RT) using intensity modulated radiation therapy (IMRT) technique, and to validate and compare the NTCP models used in RayStation. The parameters such as n, m and TD_50_ in Lyman Kutcher Burman [9] model, were fitted and compared with other published findings [10–12], in which, some of them were used in RayStation. The results will also serve to compare previous NTCP modeling in the era of 3-D and IMRT chemo-RT and offer new steps towards the ability to not only incorporate NTCP into treatment plan optimization, but also to share better defined expectations on expected treatment outcomes during consultation, on-treatment visit (OTV), and follow-up visits with patients.

## Methods

The hypothesis of this retrospective and single institution study is that the acute esophagitis was related to the dose-volume parameters, and the dose-volume parameters could be dosimetric predictors of acute esophagitis.

A total of 139 charts of patients with lung cancer who underwent chemo-RT during this period were retrieved and reviewed. Multiple chemotherapy agents were utilized and included carboplatin, cisplatin, alimta, paclitaxel, etoposide etc. This research was approved by the Institutional Review Board (IRB) of University of Mississippi Medical Center, Jackson, MS, USA. All patients with lung cancer treated with IMRT in our department between January 01, 2014 and June 30, 2017 were included in this study cohort.

All patients underwent computed tomography (CT) simulation in the supine, head first position on a wing board with both arms placed above the head. The scan was obtained with 3 mm slice thickness on a Philips Brilliance Big Bore CT with 16 multi-slice capabilities. The Philips respiratory bellows was placed around the patient’s abdomen at the level of the xiphoidal tip. A four-dimensional (4-D) CT protocol that entailed a free breathing scan was selected. In this process, a three-dimensional (3-D) standard helical scan extending from the chin to about 5 cm below the diaphragm is first obtained. This is followed by acquisition of the 4-D CT scan of the lung region that encompasses the tumor and involved lymph nodes with a 2 cm superior and 2 cm inferior margin. The isocenter was set at the time of CT simulation on the free-breathing 3-D scan using the Philips virtual simulation software. The isocenter coordinates were then sent to the LAP laser system for isocenter display on the patient’s skin where the therapist would tattoo the isocenter.

The gross tumor volume (GTV), gross nodal volume (GNV), skin, lungs, esophagus, spinal cord, and heart, etc. were contoured by the radiation oncologist on the axial view of the 3-D CT data set in Pinnacle TPS (Version 8–9.10, Philips Medical Systems, Fitchburg, WI, USA). An internal target volume (ITV) was generated from all phases of the 4-D CT image set, using the maximal intensity projection (MIP) image set. This ITV was expanded to generate a clinical target volume (CTV) encompassing subclinical disease as determined by the radiation oncologist. The CTV was then expanded by a margin of 0.5 cm to create the planning target volume (PTV). IMRT treatment plans were then generated using the Pinnacle treatment planning system (TPS).

**Fig. 1:**
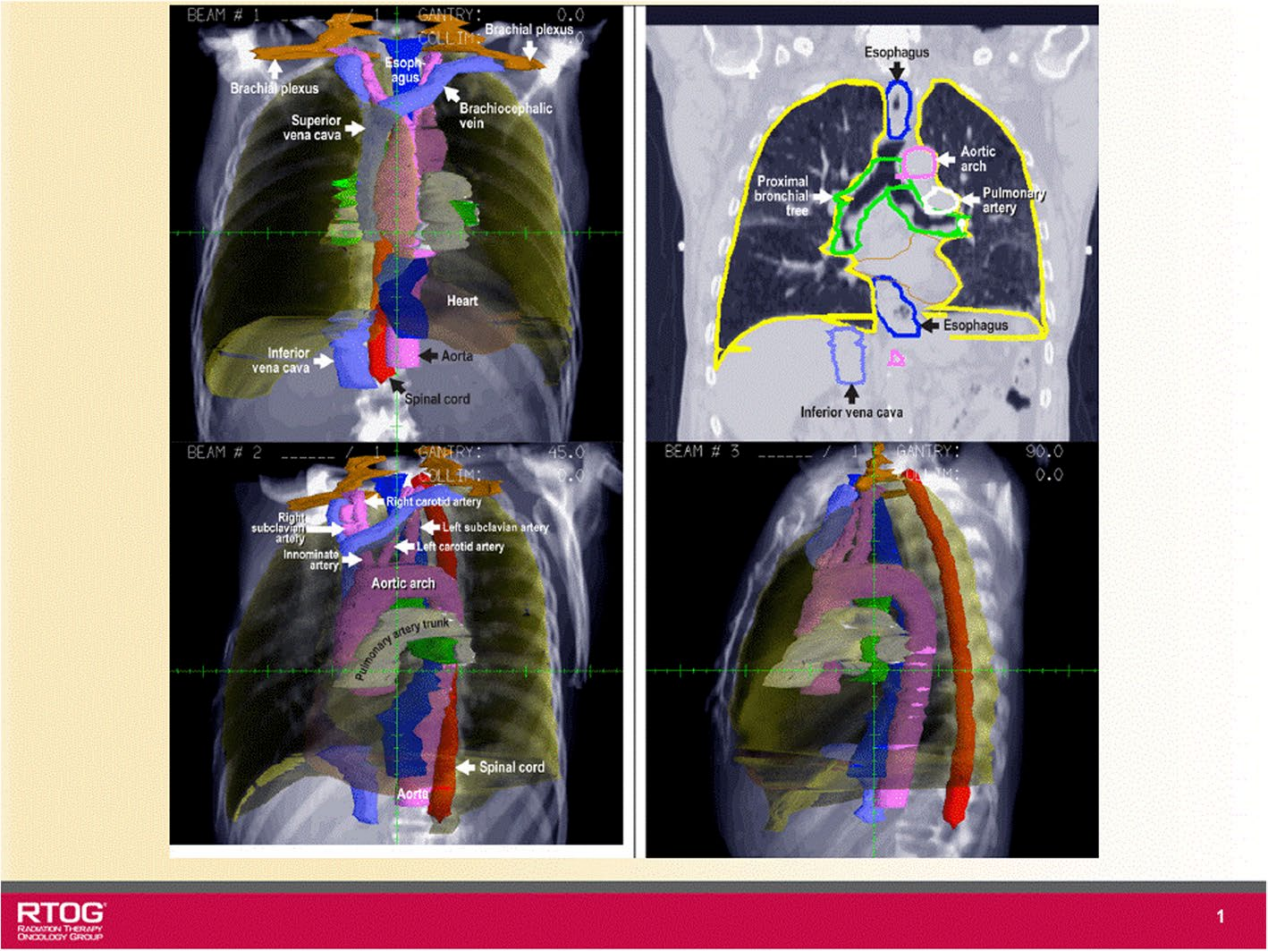
Guideline of RTOG 1106 OARs contours for lung cancer radiotherapy

The OARs were drawn based on the RTOG 1106 atlas guideline [13]. The esophageal exposure dose varied depending on the tumor location and the beam orientation selected. For current analysis, the esophagus was defined from the level of the thoracic inlet superiorly to the gastroesophageal junction inferiorly see in Fig. 1. Due to limited visibility on image data sets and mobility of the esophagus, the delineation of the esophagus can vary from one institution to another and also from one physician to another [3, 14, 15].

The treatment plan was generated using so called “Low Modulated” IMRT (LM-IMRT) on the Pinnacle TPS. With the technology development, IMRT technique has been increasingly implemented for conventional fractionated lung irradiation because of its ability to spare organs at risk such as the spinal cord, heart, esophagus and healthy lungs [16]. However fully modulated IMRT possesses unique challenges for lung cancer treatment mainly due to the interplay between lung tumor motion and small segment Multi-leaf Collimator (MLC) movements. When the trajectory of the MLC and the target is not synchronous, blurring of the dose may occur around the target [17]. LM-IMRT is a sparsely intensity modulated radiation therapy technique implemented at our institute since 2010 and used by limiting both the degree of modulation, number of segments per beam and the IMRT segment size during the inverse planning process. Ultimately, the goal of the “LM-IMRT technique is to create a hybrid plan that behaves similar to 3D CRT without small MLC segments crossing the tumor, yet providing organ sparing of an IMRT inverse plan. Our institutional guideline for this technique is to start the optimization with minimum segment size of 40 cm^2^ and minimum MU of 20 per segment; However these values can be iteratively modified. The dosimetric criterion of the plan is V_95%__Rx_ ≥ 95%, which means that the 95% of the PTV in volume should receive more than 95% of the prescription dose. In order to achieve this goal, 5–7 beams were chosen and optimally placed to avoid as many beams entering or exiting the organs at risk (OAR). In addition, ring structures and PTV optimization structures were created to achieve the desired dose coverage and gradient. Collapsed cone convolution (CCC) dose calculation algorithm and inhomogeneity correction were used for dose computation with calculation grid size of 2 mm. The LM-IMRT technique has been utilized almost exclusively at our institution since 2010 for virtually all stage II or III lung cancer patients to generate a better conformal dose distribution around tumor and give a minimum radiation dose to the surrounding healthy tissues. The total dose of 59.4—60 Gy with a daily fraction of 1.8 Gy—2 Gy using 6 MV photon beams prescribed to 95% of the PTV volume with the planning goals described above.

The MED and V_x_ of all the patients treated with LM-IMRT technique was determined from the dose volume histograms of the Pinnacle TPS, together with the endpoints of esophagitis as documented in the electronic medical record system (EMR) by our institution’s attending radiation oncologists during weekly on-treatment visits (OTV). The grade of esophagitis was based on the Radiation Therapy Oncology Group’s (RTOG) classification as shown in Table 1 and determined for each patient. The treatments were all delivered using an Elekta Synergy Linear Accelerator.

**Table 1:** RTOG classification of acute Esophagitis [3]

Grade 0	Grade 1	Grade 2	Grade 3	Grade 4
No change over baseline	Mild dysphagia or odynophagia/may require topical anesthetic or non-narcotic analgesics/may require soft diet	Moderate dysphagia or odynophagia/may require narcotic analgesics/may require puree or liquid diet	Severe dysphagia or odynophagia with dehydration or weight loss (15% from pre-treatment baseline) requiring NG feeding tube, I.V. fluids or hyperalimentation	Complete obstruction, ulceration, perforation, fistula

The MED and V_x_ for each individual lung patient were correlated with their grade 1 or 2 esophagitis using binary logistic regression and Probit statistical analysis. All the statistical analyses were performed using IBM SPSS software v. 24 at the 5% significance level (α = 0.05).The parameters of Lyman Kutcher Burman model, n, m and TD_50_, were fitted and compared with other published findings.

## Results

**Fig. 2:**
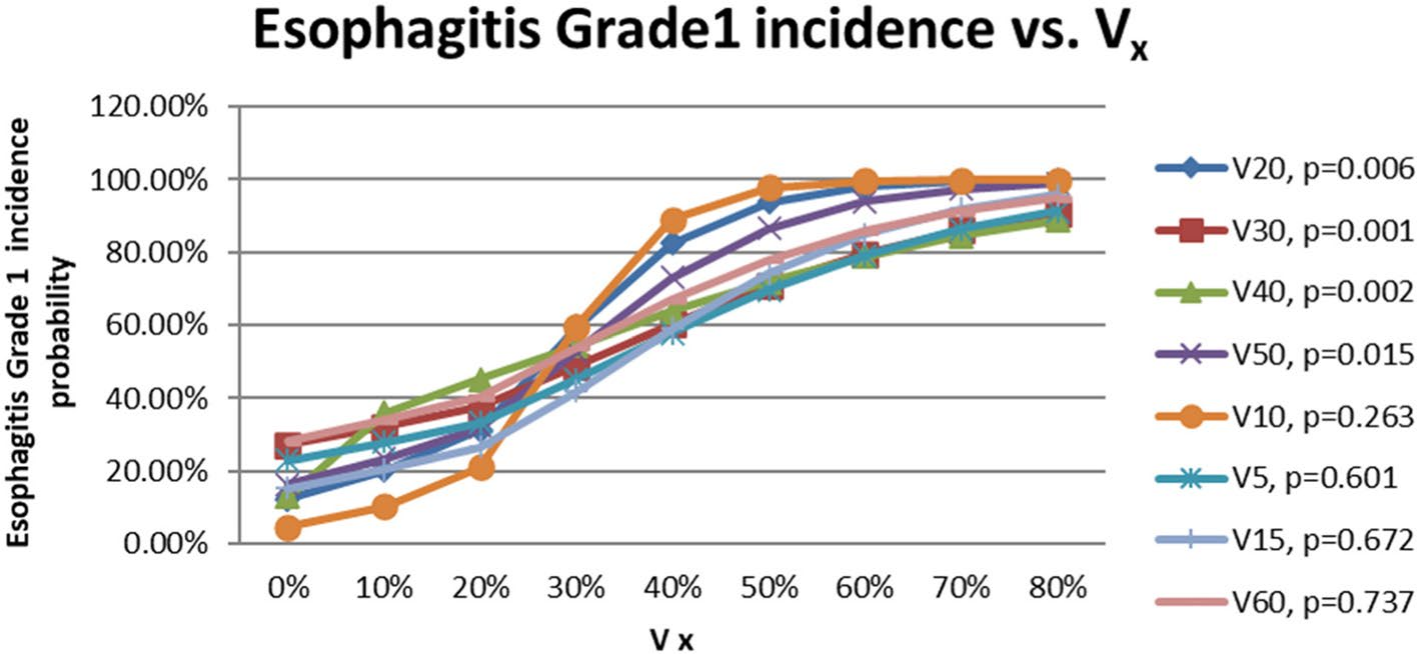
The relationships of the probabilities of incidence of esophagitis grade 1 with V_5_, V_10_, V_15_, V_20_, V_30_, V_40_, V_50_, and V_60_

**Fig. 3:**
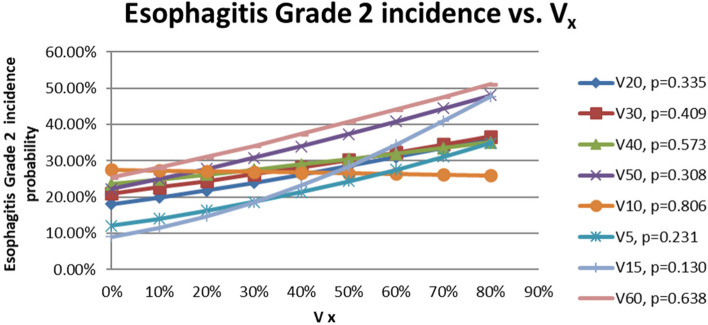
The relationships of the probabilities of incidence of esophagitis grade 1 with V_5_, V_10_, V_15_, V_20_, V_30_, V_40_, V_50_, and V_60_

**Fig. 4:**
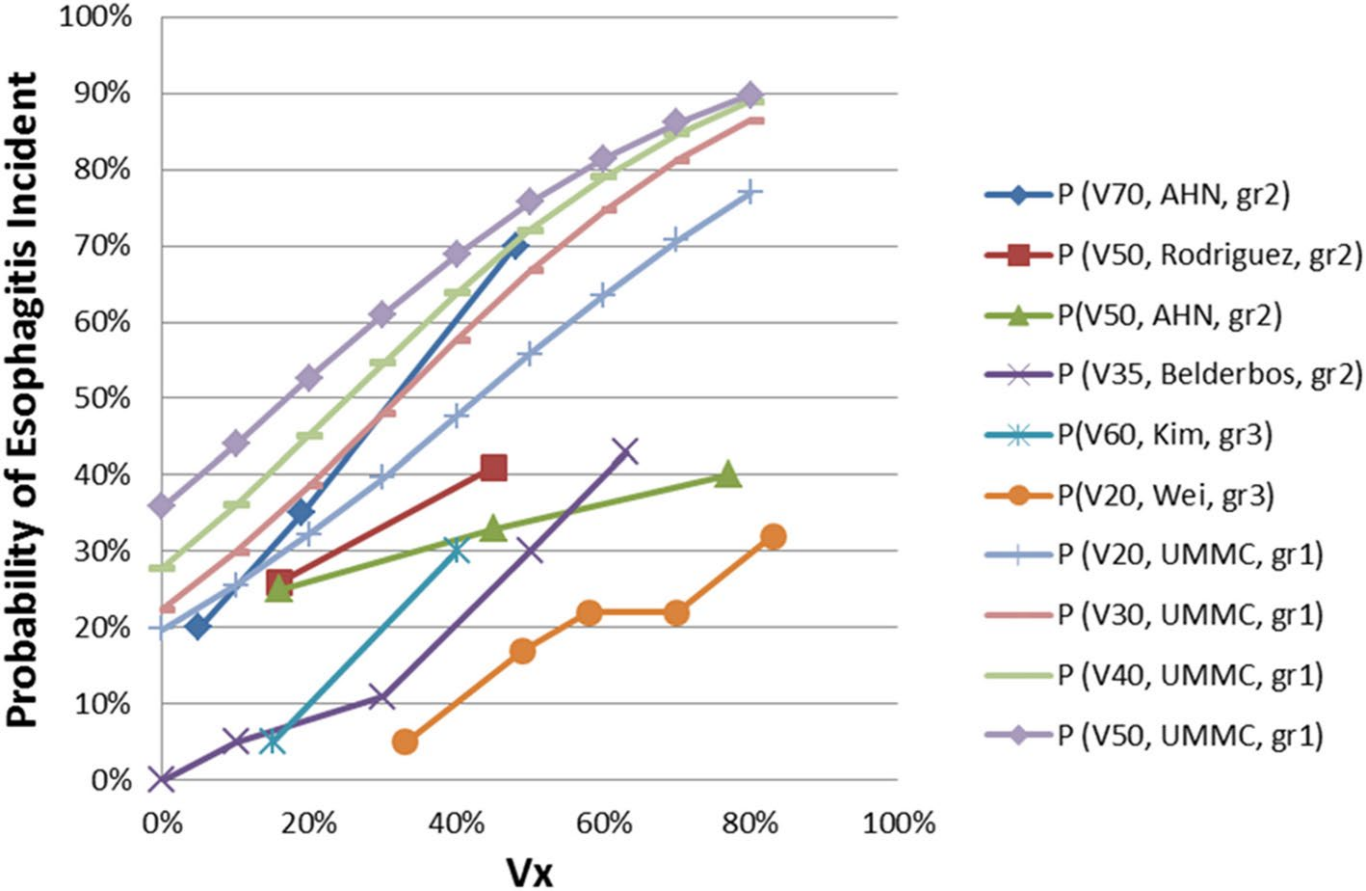
Combined the data of esophagitis grade 1 of this study to the figure in the QUANTEC paper (MARIA WERNER-WASIK, et al., 2010, S90, Fig. 2) [10]

The logistic regression relationships of the probabilities of incidence of esophagitis grade 1 and 2 with V_5_, V_10_, V_15_, V_20_, V_30_, V_40_, V_50_ and V_60_ are shown in Figs. 2 and 3. The x-axis in Figs. 2 and 3 represent the values of V_5_, V_10,_ V_15_, V_20_, V_30_, V_40_, V_50,_ and V_60_. The y-axis represents the probabilities of the incidence of esophagitis grade 1 and 2, respectively. It appeared the probabilities of incidence of esophagitis grade 1 and 2 increased with increasing of the values of V_x_. Figure 4 added some of the data of esophagitis grade 1 in this study onto the figure in the QUANTEC paper (MARIA WERNER-WASIK, et al., 2010, S90, Fig. 2) [10] for comparison and supplement purposes. It shows the general trends that acute esophagitis rate increased with increasing of the values of V_x_ for grade 1 and grade 2 of esophagitis. It can be seen as well that the slops of the trends decreased with increasing of the esophagitis grade. The lower the esophagitis grade is, the smaller the values of V_x_ are required to develop esophagitis. Since IMRT technique spared dose to OAR effectively, the dose delivered to esophagus was less, and so more esophagitis grade 1 were observed, instead of grade 2 and 3 as shown in previous findings [10]. Finally, Table 2 shows the Vx constraints added onto the QUANTEC data.

**Table 2:** Vx constraints added onto the QUANTEC data

Esophagitis			probability		*p* value
QUANTEC	MED	< 34 Gy	5–20%	Grade 3 + esophagitis	
QUANTEC	V35	< 50%	< 30%	Grade 2 + esophagitis	
QUANTEC	V50	< 40%	< 30%	Grade 2 + esophagitis	
QUANTEC	V70	< 20%	< 30%	Grade 2 + esophagitis	
This Study	MED	< 10 Gy	5–20%	Grade 2 + esophagitis	0.180
This Study	V50	< 35%	< 30%	Grade 2 + esophagitis	0.308
This Study	MED	< 300 cGy	5–20%	Grade 1 + esophagitis	0.005
This Study	V20	< 30%	< 30%	Grade 1 + esophagitis	0.006
This Study	V30	< 20%	< 30%	Grade 1 + esophagitis	0.001
This Study	V40	< 10%	< 30%	Grade 1 + esophagitis	0.002
This Study	V50	< 1%	< 30%	Grade 1 + esophagitis	0.015

Lyman-Kutcher-Burman (LKB) model is probably the most widely used for NTCP modeling in radiotherapy [9]. The three equations listed below are used in the LKB model. The model has four parameters: NTCP, TD_50_, *m* and *n*. TD_50_ represents the tolerance dose for a homogenous dose distribution to a full organ from which the risk of complications is 50%. The parameter *m* is related to the standard deviation of TD_50_ and is a measure of the slope of the sigmoid shaped dose–response curve while the parameter *n* describes the volume effect of the organ being assessed. An Equivalent Uniform Dose (EUD) is the dose at which a given uniform dose to the entire organ would produce the same NTCP as the original dose distribution by assuming that any two dose distributions are equivalent if they cause the same radiobiological effects [18].

1 $$EUD= {\left(\sum\nolimits_{i}{v}_{i}{D}_{i}^{1/n}\right)}^{n}$$

2 $$t= \frac{EUD-T{D}_{50}}{mT{D}_{50}}$$

3 $$NTCP=\frac1{\sqrt{2\pi}}\int_{-\infty}^te^\frac{-x^2}2dx$$

Equations (2) and (3) represent a sigmoidal curve that is determined by three parameters, TD_50_, *m* and *n*, which is however exactly same as probit function formula in statistical as $\Phi=\frac1{\surd2\pi}\int_{-\boldsymbol{\infty}}^{a+bX}e^{-\frac{Z^2}2}dZ$ and $\mathrm{P}={\mathrm{e}}^{\left(\mathrm{a}+\mathrm{bx}\right)}/\left({1+\mathrm{e}}^{\left(\mathrm{a}+\mathrm{bx}\right)}\right)$. Here, P is the probability. Combining probit function formula with LKB model, we derived X is the EUD, a equals to -1/m, and b equals to 1/mTD_50_ or –a/TD_50_. m or a is a measure of slope of sigmoid curve. When *n* = 1, the model reverts EUD to the mean dose (MD) of an organ. Although TD_50_ is strongly dependent on the grade of the organ toxicity, *n* is often considered as a tissue characteristic. The MD model is widely used due to its simplicity and effectiveness. It was the metric used for the large multi-institutional analysis and often performs as well as the more complex model [14].

**Fig. 5:**
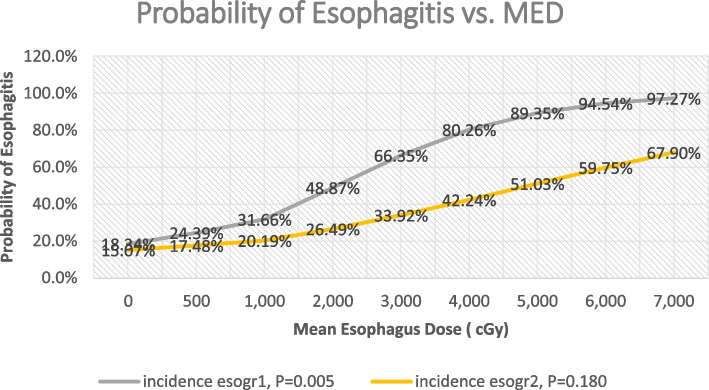
Probability of esophagitis vs. mean esophagus dose for both grades

**Fig. 6:**
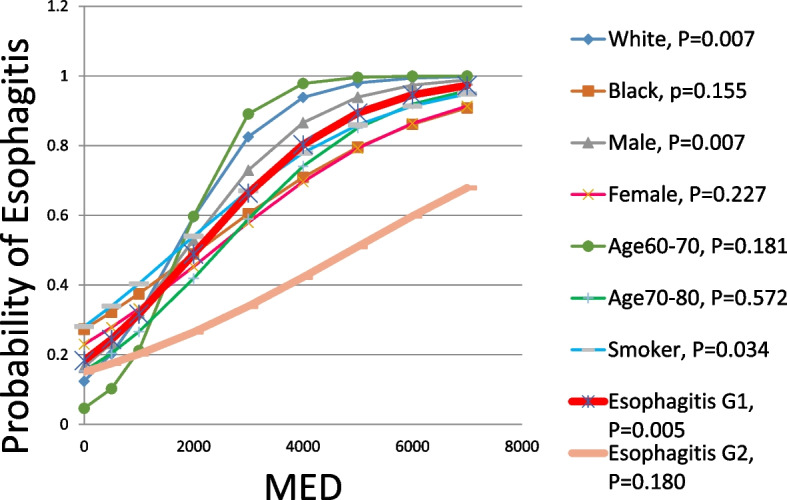
Logistic regression of probability of esophagitis grade 1 vs. MED in respect to races, gender, age and smoker

Figure 5 shows a logistic regression of probability of esophagitis grade 1 and 2 vs. MED. The general trend is that the esophagitis incidence probability increased with increasing MED. This quantitatively shows that the MED is a useful predictor for the incidence of esophagitis (grade 1 and 2). It reveals that a typical sigmoid shape of the probability of esophagitis grade 1 vs. MED, with *m* as the slope of the curve times TD_50_ being determined as 0.237. By transferring the sigmoid dose response curve of esophagitis grade 1 to a straight line using probit analysis, the TD_50_ of the incidence of esophagitis grade 1 is determined as 1510 cGy. However, esophagitis grade 2 vs. MED does not show the sigmoid dose response curve but shows a gradual increase in dose response. Even though, m for grade 2 of esophagitis was determined as 0.395. By transferring the dose response curve of esophagitis grade 2 to a straight line using probit analysis, the TD_50_ of the esophagitis grade 2 is determined as 4594 cGy. Furthermore, TD_30_ and TD_20_ of esophagitis grade 2 were determined as 1748 cGy and 974 cGy respectively. Moreover, the plots of the probability of esophagitis versus MED demonstrated a dose response phenomenon, suggesting that there may be no absolute “safe” threshold MED below which there is no esophagitis [19]. The treatment planning constraints on the acceptable MED and the risk of esophagitis in each patient will depend on the individual risk/benefit ratio as determined by the clinician [19]. Furthermore, Fig. 6 shows the sigmoid shaped dose responding curve between probability of esophagitis grade 1 and MED in respect to races, gender, age group, smokers. It can be seen that the white smoker males have the higher probability to develop the esophagitis grade 1 when their lung cancer were treated using chemo-RT.

The comparison between our results (bold in the Table 3) and previous results from three publications regarding the LKB model parameters was listed in Table 3. It can be seen that the TD_50_ of 68 Gy (Burman et al., 1991) [9] is quite different compared to the values of our institute and other two investigators. This could be because the endpoint in their findings considered very severe toxicity due to non-advanced treatment techniques in the year of 1991. However, our findings of m and TD_50_ for esophagitis grade 2 agreed with other two investigators (Chapet et al., 2005 [11] and Belderbos et al., 2005 [12]). These results elucidate that the implementation of IMRT treatment technique improved the dosimetric parameters of esophagus which is evident in the infrequency with which this patient population experienced grade 2 esophagitis as our model agrees with others. Additionally, this work offers additional clinical insight in the creation of an NTCP model for grade 1 esophagitis which can require some medical care.

**Table 3:** Comparison to previously reported results of the LKB model parameters

Investigator	TD_50_ (Gy)	n	m
Burman et al., 1991 [9]	68	0.06	0.11
Chapet et al., 2005 [11] (Esophagitis Grade 2 or Greater)	51 (20–82)	0.44 (0.11–0.41)	0.32 (0.19–0.57)
Belderbos et al., 2005 [12] (Esophagitis Grade 2 or Greater)	47 (45–60)	0.69 (0.18–6.3)	0.36 (0.25–0.53)
**This study, 2017 (Esophagitis Grade 1) IMRT**	**15.1 (10.6–19.6)**	**1**	**0.237**
**This study, 2017 (Esophagitis Grade 2) IMRT**	**45.9 (33.9–71.4)**	**1**	**0.395**

In consideration of the desire to utilize this information directly in treatment planning, our model was utilized in retrospective radiobiological evaluation of the treatment plan in RayStation TPS for one patient who developed grade 1 and 2 esophagitis. Our grade 1 model applied to the dose distribution predicted an 86% chance of grade 1 esophagitis while the grade 2 models only predicted 7% chance of grade 2 esophagitis (see Table 4). This demonstrates the feasibility of incorporation of these types of models into the treatment planning process, but also the need for further work to validate these models in the clinical setting beyond the lab. To make these models valid clinically for specific patients, likely other factors besides just dosimetry will likely need to be considered. Future work will look to improve these models by direct application to patient dose distributions and correlation with clinical toxicity outcomes for potential improvement.

**Table 4:**
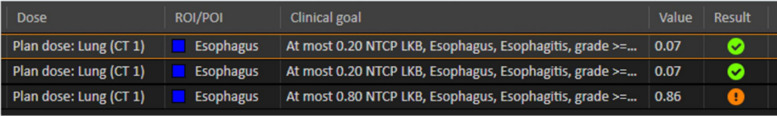
Retrospective radiobiological evaluation of the treatment plan in RayStation TPS for one patient who developed both esophagitis grade 1 and grade 2

## Discussion

The purpose of treatment planning is to design or optimize the radiation beams that maximizes the tumor control probability (TCP) and minimizes normal tissue complication probability (NTCP). With conventional IMRT optimization in most of treatment planning systems, the treatment goal is indirectly achieved by the dose volume histograms (DVH) distribution to the tumor and OARs based on objective functions that do not represent the nonlinear response of tumor or OARs to dose adequately [6]. RaySearch seeks to be more direct in their treatment planning system, RayStation, through direct biological evaluation and even optimization within a module called RayBiology. Given a treatment plan, a radiobiological index such as TCP and NTCP is calculated for each organ of interest [8] to capture what happens when tumor and OARs are irradiated based on the best clinical treatment outcome data. In order to utilize such functionality reliably, models must be validated independently and additional confounding factors be understood. This study established the relationships between esophagitis (grade 1 and grade 2) with V_x_ and MED for lung cancer radio-chemotherapy using LM-IMRT treatment technique. The fitted LKB model in this study is used to compare and validate the previous models used in RayStation with some degree of limitations.

The first limitation of this study is the patient number which may be small, especially for esophagitis grade 2 which includes only 32 patients as shown in Table 5. That is probably why NTCP models of esophagitis grade 2 in this study is not significant (*p* > 0.05) using V_x_ and MED. In contrast, *p* value is smaller than 0.05 for esophagitis grade 1 with which 58 patients have developed. Table 5 displays the frequency distribution of esophagitis grade 1 and grade 2, respectively. It can be seen that out of total 139 patients, there were 58 (41.7%) patients who developed the esophagitis grade 1 (G1), and 32 (23.0%) patients who developed esophagitis grade 2 (G2), and 34 (24.5%) patients who did not develop esophagitis (G0) during the treatment course. 15 patients without on-treatment notes about esophagitis grades, were excluded, leaving 124 evaluable patients for the related statistical tests. The number of patients with esophagitis grade 0 is calculated as 139–15-58–32 = 34.

**Table 5:** Frequency distribution of esophagitis grade 0, 1 and 2

**Esophagitis – Grade 1**					
		Frequency	Percent	Valid Percent	Cumulative Percent
Valid	no	66	47.5	53.2	53.2
	yes	58	41.7	46.8	100.0
	Total	124	89.2	100.0	
Missing	System	15	10.8		
Total		139	100.0		
**Esophagitis – Grade 2**					
		Frequency	Percent	Valid Percent	Cumulative Percent
Valid	no	92	66.2	74.2	74.2
	yes	32	23.0	25.8	100.0
	Total	124	89.2	100.0	
Missing	System	15	10.8		
Total		139	100.0		

Secondly, clinical data indicate that both patient-related and treatment-related factors contribute to normal esophageal toxicities [20]. A lot of risk factors such as height, weight, gender, age, personal health condition, cancer stage, esophagus length, treatment technique, dosimetric parameters, dose per fraction, overall treatment time, volume of tumor/normal tissue irradiated, concomitant use of chemotherapy etc. are correlated to treatment outcomes [2]. In this study we only focused on and limited on dosimetric parameters using IMRT treatment technique.

The third limitation is the difficulty in defining the volume of esophagus in radiation therapy. The esophagus is a tube organ and remains closed when no swallowing activities. Its lumen is often difficult to be determined, particularly in the middle and caudal levels. Clinically, a thick barium paste is administrated to help to localize the esophagus in CT image but the barium paste might cause the uncertainty of the dose calculation due to the high electronic density of the barium paste. The esophagus is slightly moving, and the cephalad, middle and caudal esophagus can move up to 5, 7, and 9 mm in the combined anterior–posterior and cephalad-caudal directions, respectively [21], that could cause some inaccuracies in dose-volume calculations using the planning CT scan. There are no specific margin recommendations given yet. The dose-volume histogram (DVH) is only a sample of a possible scenario of the esophagus dose during treatment and does not accurately represent the actual partial volume doses due to the esophagus variation during swallowing activity [22].

## Conclusions

In this study, a dosimetric predictive model for grade 1 and 2 esophagitis in lung cancer patients treated with Chemo-RT using LM-IMRT technique was established. This model also compared to previous models based on the early 3D data. We found that the mean esophagus dose (MED) and V_20_, V_30_, V_40_ and V_50_ are statistically useful predictors of radiation induced esophagitis amongst other demographic factors. The TD_50_ of grade 1 and 2 esophagitis were determined. In addition, all the data for this study including consistent contouring of OARs, specific planning technique, daily imaging guidance in treatment, and outcome data is from a single institution that minimized the variability in volumetric metrics of the esophagus arising from the combining of data from multiple institutions. Studies like this one are invaluable as additional clinical guidelines in push towards not only radiobiological treatment plan evaluation but also optimization. The movement towards this will improve the process of counseling the patient on the risks versus benefits of lung radiation therapy in addition to the ability to be medically prepared for probable outcomes.

## Data Availability

The datasets used during the current study are available from the corresponding author on reasonable request.

## Abbreviations

3-D: Three-dimensional
4-D: Four-dimensional
AAPM: The American association of physicists in medicine
Chemo-RT: Chemoradiotherapy
CT: Computed tomography
CTV: Clinical target volume
DVH: Dose Volume Histogram
EMR: Electronic medical record system
EUD: Equivalent Uniform Dose
GNV: Gross nodal volume
GTV: Gross tumor volume
IMRT: Intensity modulated radiation therapy
IRB: Institutional Review Board
ITV: Internal target volume
LKB: Lyman-Kutcher-Burman
MED: Mean esophagus dose
MD: Mean dose
MIP: Maximal intensity projection
NTCP: Normal tissue complication probability
OAR: Critical organs at risk
OTV: On-treatment visit
PTV: Planning target volume
QUANTEC: Quantitative Analysis of Normal Tissue Effects in the Clinic
RT: Radiation therapy
RTOG: Radiation Therapy Oncology Group
TCP: Tumor control probability
TPS: Treatment planning system
UMMC: University of Mississippi Medical Center
